# Pharmaceutical targeting Th2-mediated immunity enhances immunotherapy response in breast cancer

**DOI:** 10.1186/s12967-022-03807-8

**Published:** 2022-12-23

**Authors:** Yuru Chen, Jiazheng Sun, Yachan Luo, Jiazhou Liu, Xiaoyu Wang, Rui Feng, Jing Huang, Huimin Du, Qin Li, Jinxiang Tan, Guosheng Ren, Xiaoyi Wang, Hongzhong Li

**Affiliations:** 1 Chongqing Key Laboratory of Molecular Oncology and Epigenetics, The First Affiliated Hospital of Chongqing Medical University, Chongqing, 400016 China; 2 Department of Endocrine and Breast Surgery, The First Affiliated Hospital of Chongqing Medical University, Chongqing, 400016 China; 3 Department of Pharmacy, The First Affiliated Hospital of Chongqing Medical University, Chongqing, 400016 China; 4 Department of Respiratory, The First Affiliated Hospital of Chongqing Medical University, Chongqing, 400016 China; 5 Department of Oncology, The First Affiliated Hospital of Chongqing Medical University, Chongqing, 400016 China; 6 Department of Oncology, Beijing Friendship Hospital, Capital Medical University, Beijing, 100050 China

**Keywords:** Th2 cell, Suplatast tosilate, Breast cancer, Immune checkpoint blockade, Immunotherapy

## Abstract

**Background:**

Breast cancer is a complex disease with a highly immunosuppressive tumor microenvironment, and has limited clinical response to immune checkpoint blockade (ICB) therapy. T-helper 2 (Th2) cells, an important component of the tumor microenvironment (TME), play an essential role in regulation of tumor immunity. However, the deep relationship between Th2-mediated immunity and immune evasion in breast cancer remains enigmatic.

**Methods:**

Here, we first used bioinformatics analysis to explore the correlation between Th2 infiltration and immune landscape in breast cancer. Suplatast tosilate (IPD-1151 T, IPD), an inhibitor of Th2 function, was then employed to investigate the biological effects of Th2 blockade on tumor growth and immune microenvironment in immunocompetent murine breast cancer models. The tumor microenvironment was analyzed by flow cytometry, mass cytometry, and immunofluorescence staining. Furthermore, we examined the efficacy of IPD combination with ICB treatment by evaluating TME, tumor growth and mice survival.

**Results:**

Our bioinformatics analysis suggested that higher infiltration of Th2 cells indicates a tumor immunosuppressive microenvironment in breast cancer. In three murine breast cancer models (EO771, 4T1 and EMT6), IPD significantly inhibited the IL-4 secretion by Th2 cells, promoted Th2 to Th1 switching, remodeled the immune landscape and inhibited tumor growth. Remarkably, CD8^+^ T cell infiltration and the cytotoxic activity of cytotoxic T lymphocyte (CTL) in tumor tissues were evidently enhanced after IPD treatment. Furthermore, increased effector CD4^+^ T cells and decreased myeloid-derived suppressor cells and M2-like macrophages were also demonstrated in IPD-treated tumors. Importantly, we found IPD reinforced the therapeutic response of ICB without increasing potential adverse effects.

**Conclusions:**

Our findings demonstrate that pharmaceutical inhibition of Th2 cell function improves ICB response via remodeling immune landscape of TME, which illustrates a promising combinatorial immunotherapy.

**Supplementary Information:**

The online version contains supplementary material available at 10.1186/s12967-022-03807-8.

## Introduction

Breast cancer is a complex disease associated with high morbidity and mortality rates [1]. It is classified into triple-negative breast cancer (TNBC), hormone receptor positive (HR +) with estrogen (ER) and/or progesterone (PR) receptor, and human epidermal receptor 2 positive (HER2 +) [2]. Compared with HR + subtype, which is characterized by low tumor-infiltrating lymphocytes (TILs) infiltration, TNBC and HER2 + subtypes are associated with high infiltration of TILs [3, 4]. Notably, higher TIL levels are associated with improved prognosis and decreased risk of relapse and death [5, 6]. Immune checkpoint blockade (ICB) therapies targeting programmed death 1 (PD-1), programmed death ligand 1 (PD-L1), and cytotoxic T lymphocyte-associated antigen 4 (CTLA-4) have produced enormous clinical efficacy in multiple cancer patients [7]. Increasing studies exhibit that ICB has a clinical response against advanced breast cancer [8–10]. Unfortunately, only a small proportion of patients (less than 20%) with breast cancer can get benefit from ICB therapy [8]. Thus, how to surmount the resistance to immunotherapy and improve clinical efficacy in breast cancer is worth further exploration.

Multiple factors are correlated with therapeutic efficacy of ICB in breast cancer, including the degree of cytotoxic T lymphocyte (CTL) infiltration, tumor mutation burden, the expression level of immune checkpoint and immunosuppressive factors in tumor microenvironment (TME) [9, 10]. Increasing evidence indicates that immunological composition and functional status of TME have a critical role in ICB resistance [10, 11]. The infiltration of immune cells, such as T cell, myeloid-derived suppressor cell (MDSC), and tumor-associated macrophage (TAM) in TME, can affect therapeutic efficacy of ICB therapy. Tumor cells can co-evolve with immune cells and evolve a variety of strategies to escape immune destruction [12–14]. T-helper 2 (Th2) cell, a vital component of TME, plays a central role in type-2 immune responses (“humoral immunity”) and up-regulates antibody production to fight extracellular tissues [15, 16]. Th2 cell-derived cytokines including interleukin-4 (IL-4), IL-5, and IL-13 underlie the inappropriate immune response, and lead to anaphylactic diseases such as asthma, chronic rhinitis, atopic dermatitis, and certain types of gut disorders [17–19]. It is indicated that Th2 cell plays a controversial role in cancer development. It is shown that high infiltration of Th2 cell could cause the epigenetic reprogramming of tumor cells and suppress breast tumorigenesis [20]. Additionally, Th2 cells are positively involved in tumor regression by triggering an inflammatory immune response [21]. In contrast, several lines of evidence support that the initiation of local Th2 inflammation could foster an immunosuppressive microenvironment and aid tumor progression [22–24]. Therefore, the relationship between Th2 and immune evasion in breast cancer remains enigmatic, which needs further in-depth investigation.

Allergies are abnormal, Th2-biased immune responses against innocuous environmental antigens [25]. Our previous study found that the allergy mediator histamine could suppress tumor growth, and induce immunotherapy resistance via binding to histamine receptor H1 on macrophages, and anti-histamine therapy could synergistically enhance efficacy of ICB therapy [26]. However, anti-histamine only partially reversed allergy-induced immunotherapy resistance. Regarding Th2-mediated immunity is closely implicated in allergic diseases, we supposed that it might also contribute to the treatment resistance. In addition, the shift in Th1 and Th2 skewing and Th2 cytokine secretion vary after ICB therapy in a context-dependent manner in cancer [27]. Therefore, we sought to further explored whether targeting Th2-mediated immunity could have synergistic effects with ICB therapy. Suplatast tosilate (IPD-1151 T, IPD) was used for curing anaphylactic diseases including allergic rhinitis, atopic dermatitis, and bronchial asthma [28]. Multiple studies indicate that IPD is deemed as an inhibitor of targeting Th2-mediated immunity and can suppress IL-5 and IL-4 secretion from Th2 cells [29, 30]. Here, we employed IPD to test whether targeting Th2-mediated immunity can influence immune landscape and therapeutic efficacy of immunotherapy in breast cancer.

## Materials and methods

Additional Materials and Methods can be found in online Additional files (Additional file 9).

### Bioinformatics analyses

The Cancer Genome Atlas (TCGA) and Molecular Taxonomy of Breast Cancer International Consortium (METABRIC) gene expression data and clinicopathology data were extracted from cBioPortal (http://www.cbioportal.org/) [31], and other breast cancer cohorts’ data was accessed from Gene Expression Omnibus (GEO) datasets (https://www.ncbi.nlm.nih.gov/geo/). We reanalyzed public datasets to show the functional gene expression signatures (Fges) scores (http://science.bostongene.com/tumor-portrait/). Breast cancer was screened based on immunohistochemistry (ER, PR and Her2). The correlation analysis between GATA3 expression and CTL infiltration in 9 breast cancer datasets was administrated on Tumor Immune Dysfunction and Exclusion (TIDE) system (http://tide.dfci.harvard.edu/faq/) [32]. CIBERSORT algorithm was utilized to analyze 22 types of immune cells in breast cancer tissues, including 7 T cell subtypes, memory and naïve B cells, natural killer cells, and myeloid cells. After quality filtering (p < 0.05), breast cancer samples were included in the analysis. Additionally, six immune cell infiltration including CD8^+^ T cells, CD4^+^ effector T cells, B cells, macrophages, neutrophils, and dendritic cells were analyzed by Tumor Immune Estimation Resource (TIMER) (http://timer.cistrome.org/) [33]. MTABRIC breast cancer samples were used to asses correlating Fges in TME. Signature scores of TME were calculated by python implementation of the single-sample gene set enrichment analysis (ssGSEA), and the correlation analysis of signature was performed by pearson’s correlation [34]. Th2 cell groups in breast cancer samples were based on 2 separations using GATA3 expression. CD8^+^ CTL function was elucidated using a 15-gene signature (IL2, CD8A, CCL5, GZMA, PRF1, IFNG, PTPRC, GZMM, RAB27A, XCL1, ICOS, TBX21, GZMB, GNLY, and IL12A). To test the association between GATA3 gene expression level and patient overall survival, Kaplan–Meier survival analysis was conducted based on TIDE online. T cell dysfunction scores of Th2 cells in breast cancer were evaluated by TIDE.

### Cell lines and culture

MDA-MB-231 cell (human breast cancer cell line), EO771 cells (a C57BL/6 murine breast cancer cell line), EMT6 cells (a BALB/c murine breast cancer cell line), and 4T1 cells (a BALB/c murine breast cancer cell line) were obtained from the American Type Culture Collection. All cells were cultured in RPMI-1640 (Roswell Park Memorial Institute-1640) medium with 10% FBS.

### Mice and in vivo tumor studies

For EMT6, 4T1 and EO771 models, BALB/c or C57BL/6 mice were injected respectively with 1.5 × 10^5^ or 2 × 10^5^ tumor cells orthotopically. Mice were intragastrical administered with PBS or IPD (S2015, Selleck; 100 mg/kg) at day four post tumor induction [35]. Anti-CTLA4 antibody (BioXCell, Clone:9D9, Catalog #: BP0164; 5 mg/kg) and anti- PD-1 antibody (BioXCell, Clone:29F.1A12, Catalog #: BP0273; 2 mg/kg) were administered intraperitoneally on days 7, 10, 13 after tumor inoculation. Tumor volume was measured 2 or 3 times per week by using electronic calipers, and calculated according to the following formula: volume = (length × width^2^)/2. Female BALB/c and C57BL/6 mice between 6 and 8 weeks old were applied in all experiments, and all mice were supplied by the Laboratory Animal Centre of Chongqing Medical University.

### Preparation of single-cell suspension from tumors

Tumor-bearing mice were sacrificed after tumor inoculation (EO771 and EMT6: 18 days, 4T1: 30 days). Tumor samples were harvested, chopped with scissors, and incubated with 2 mg/ml collagenase A (Roche) for 40–50 min at 37 ℃. The dissociated cells were filtered through 70 μm filters (BD Biosciences) to obtain single-cell suspensions. Afterward, erythrocytes were lysed with red blood cell lysis buffer for 1 min on ice, and single-cell suspension was washed and re-suspended with DMEM or PBS depending on further use.

### Flow cytometry

Cells were stained for Live/Dead with Fixable Viability Dye eFluor 450 (eBioscience) for 30 min at 4 ℃. After being washed once with flow cytometry buffer, cells were stained with dilutions of various combinations of primary antibodies to cell surface marker, including CD45 (APC-CY7), CD11b (BV510), CD3 (FITC), CD4 (PE-CY5.5), CD8 (PE-CY7/FITC), Gr-1 (PE), F4/80 (APC/BV605), Tim-3 (PE-CY7), PD-1 (PerCP-CY5.5) and I-A/I-E (PerCP-CY5.5). After that, cells were washed with flow cytometry buffer. Intracellular staining was administrated according to the manufacturer’s protocol. Briefly, cells were first fixed and permeabilized using the Foxp3 staining buffer kit (eBioscience), and then incubated with fluorochrome-conjugated antibodies to CD206 (APC), FOXP3 (FITC) and Ki67 (PE) from BioLegend. For intracellular cytokine staining, cells were stimulated with Cell Stimulation Cocktail (eBioscience) for 4 h at 37 ℃. Cells were then stained with anti-IFN-γ (APC) and anti-TNF-α (PE/Cyanine7). The dilution ratios for all antibodies were 1:100. After all staining procedures, cells were acquired using BD FACS Canto II and BD FACSDiva software (BD Biosciences). FlowJo software was used in subsequent analysis. The representative gating strategy for the flow cytometry was shown in the Additional file 8: Figure S8.

### Mass cytometry (CyTOF) and data analysis

Mouse tumor tissues were mechanically processed and digested as described above. After filtration using 70 μm (BD Biosciences), cells were incubated with 25 mM cisplatin for 1 min (viability staining) and then stained with mAb cocktails against intracellular proteins. Metal tagged antibodies utilized in mass cytometry analysis were purchased from Fluidigm. Cells suspension was diluted to approximately 10^6^ cells per ml using ddH_2_O containing bead standards and then analyzed on a CyTOF 2 mass cytometer (Fluidigm). CyTOF data was normalized and manually gate using Cytobank software. After CD45^+^ immune cells gated, data were transformed using cytofAsinh function before applying it to the downstream analysis. The immune subsets were generated during Phenograph clustering analysis through R cytofkit package. FlowSom, an unsupervised automated algorithm, was used to order cells based on their phenotypic similarities.

Based on the mean value of each marker in clusters, heatmaps were generated. Cell frequency of each cluster was calculated as a percent of each cell type divided by the total CD45^+^ cells in same sample. Antibodies used in the mass cytometry analysis were purchased from Fluidigm: 89Y-anti-CD45, 175Lu-anti-CD4, 141Pr-anti-PD1, 143Nd-anti-CD11b, 144Nd-anti-Siglec F, 145Nd-anti-CD69, 146Nd-anti-CD206, 148Nd-anti-Tbet, 114Sn-anti-CD103, 151Eu-anti-CD68, 152Gd-anti-CD3e, 156Gd-anti-CD14, 159 Tb-anti-F4/80, 160Dy-anti-CD62L, 161Dy-anti-Ki67, 162Dy-anti-Ly-6C, 165Ho-anti-Foxp3, 149Sm-anti-CD19, 167Er-anti-GATA3, 142Ce-anti-NK1.1, 168Er-anti-CD8a, 172Yb-anti-CD86, 173Yb-anti-CD117, 174Yb-anti-ly-6G/Ly-6C(Gr-1), 209Bi-anti-I-A/I-E, 150Sm-anti-CD11c, 169Tm-CTLA4.

### Immunofluorescence (IF) staining

Standard IF staining was performed as described previously [36]. The primary antibodies used for IF staining includes anti-granzyme B (ab4059, Abcam, 1: 800), and anti-CD8 (ab22378, Abcam, 1: 200); DyLight 488- or DyLight 594-conjugated secondary antibodies against rabbit or mouse IgG were purchased from Thermo Fisher Scientific.

### Statistical analysis

Flow cytometric data was analyzed using FlowJo software (v10; Tree Star). GraphPad Prism (v8) was used to generate graphs and for statistical analysis. Student’s t test and ANOVA were used to determine statistical significance. *P*-value < 0.05 was considered statistically significant. All the above analysis methods and R package were implemented by R foundation for statistical computing (2020) version 4.0.3 and software packages ggplot2 and pheatmap. Sanguini diagram was built based on R software package ggalluval. All the above analysis methods and R package were implemented by R foundation for statistical computing (2019) version 4.0.3. *P* < 0.05.

## Results

### Distinct immune profiling in Th2-high and Th2-low breast cancer samples

**Fig. 1:**
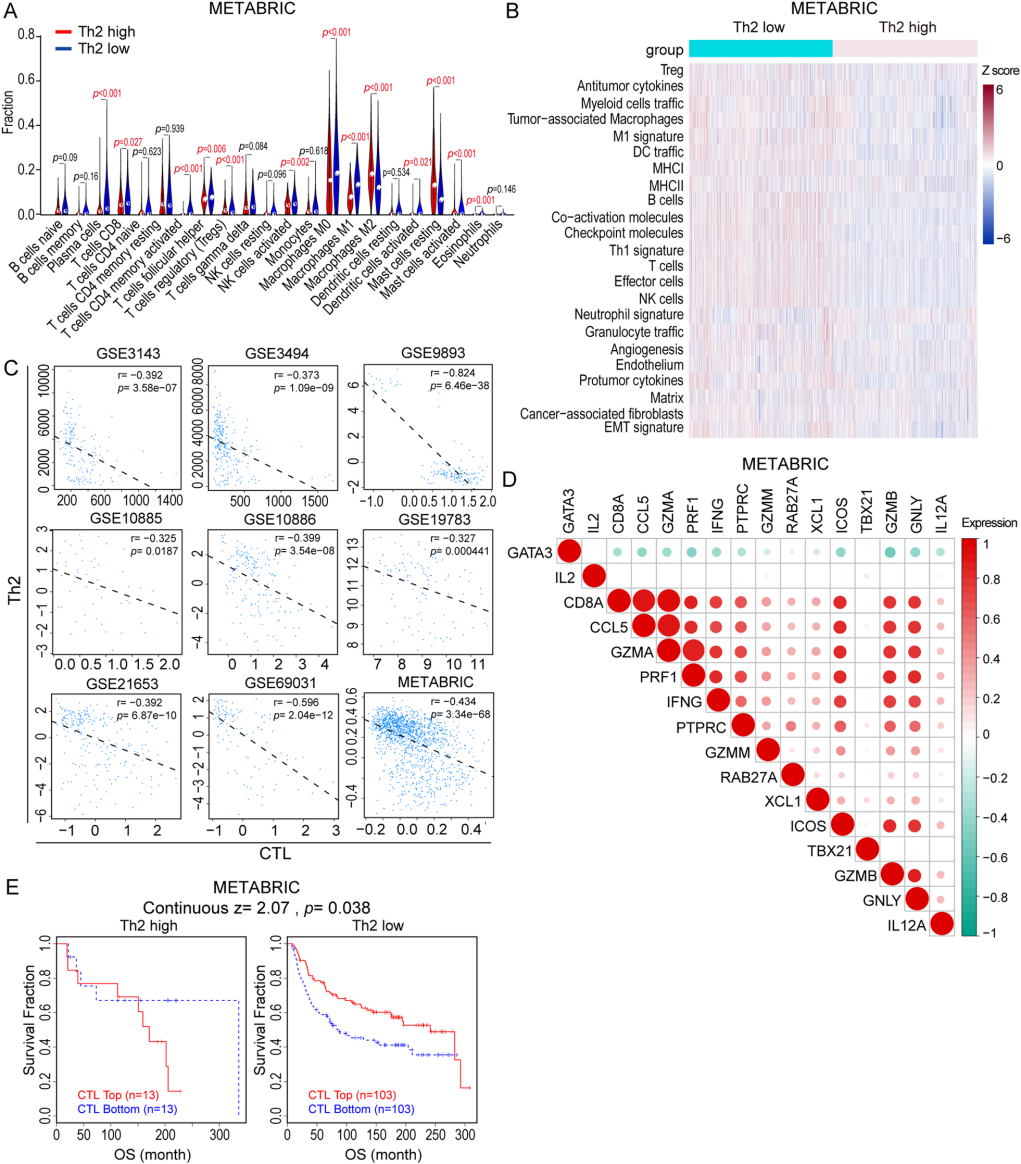
Distinct immune profiling in Th2-high and Th2-low breast cancer samples. **A** The correlation between Th2 cells and 22 types of stromal cells from TME in METABRIC breast cancer database assessed by CIBERSORT (Th2 level divided by GATA3 median level). **B** Heatmap of METABRIC database samples classified into Th2-high and Th2-low group with unsupervised clustering of the 29 FGES. **C** Pearson correlation analysis of the relationship between Th2 cell infiltration and CTL infiltration in cancer tissues from TNBC patients based on 9 BRCA datasets. **D** Correlation between Th2 signature gene (GATA3) expression and T cell function signature genes expression in breast cancer based on METABRIC database. **E** The Kaplan–Meier survival analysis of METABRIC breast cancer patients in Th2-high and -low groups based on CTL infiltration level. High CTL infiltration, CTL-Top; Low CTL infiltration, CTL-Bottom

GATA3 conducts as a master transcription factor for the differentiation of Th2 cells to activate Th2 cytokine expression in IL-4 dependent or independent pathway [37], and has been used to define Th2 cell population [38].To investigate the correlation between Th2 cells and tumor-immune landscape, we first divided the METABRIC samples into Th2-high and Th2-low groups based on GATA3 expression, and delineated the pattern of TILs in the two groups based on gene expression profile with CIBERSORT algorithm. Compared with Th2-high group, the proportions of antitumor cell population including CD8^+^ T cells, activated CD4^+^ memory T cells, M0/M1 macrophages, follicular helper T cells, and activated natural killer (NK) cells significantly increased in Th2-low group. In contrast, the proportions of suppressive immune cells including regulatory T cells (Tregs) and M2 macrophages were noticeably decreased in Th2-low group (Fig. 1A). Similarly, it also showed that antitumor immune cells such as B cells, CD8^+^ T cells, CD4^+^ effector T cells and dendritic cells were enriched in Th2-low group based on TCGA breast cancer database (Additional file 1: Fig. S1A). Furthermore, we classified breast cancer TME using curated list of Fges, which characterized the presence of immune-active or immunosuppressive microenvironment and tumor stroma. Their expression patterns were evaluated across breast cancer (METABRIC) based on ssGSEA scores. Our analysis revealed that breast cancer patients were clustered into Th2-high and Th2-low groups, which had varied significantly in immune landscape based on the expression of Fges. The Th2-low group demonstrated active immune microenvironment compared with Th2-high group (Fig. 1B). Next, we found that Th2 cells were negatively correlated with CTL infiltration in multiple breast cancer datasets based on TIDE system (Fig. 1C). To better understand the correlation among Th2 cell and CTL function, we calculated 15 genes signature expression of CTLs. The result showed that Th2-high versus Th2-low group had a different expression pattern of genes which were involved in CTLs function on METABRIC database. Clearly, GATA3 was negatively correlated with CTL signature gene (IL2, CD8A, CCL5, GZMA, PRF1, IFNG, PTPRC, GZMM, RAB27A, XCL1, ICOS, TBX21, GZMB, GNLY, and IL12A) in breast cancer (Fig. 1D). Importantly, based on TIDE system, we found that the breast cancer patients with high CTL infiltration (CTL-Top) had prolonged survival compared with low CTL infiltration (CTL-Bottom) in Th2-low group, which was not recapitulated in Th2-high group (Fig. 1E). This observation suggested that Th2 cells had positive association with T cell dysfunction, which was further supported by TCGA database (Additional file 1: Fig. S1B). Taken together, all these data demonstrated that Th2 cell was closely correlated with immunosuppressive immune landscape in breast cancer.

### Blocking Th2-mediated immunity promotes Th2 to Th1 switch and inhibits tumor growth in breast cancer

**Fig. 2:**
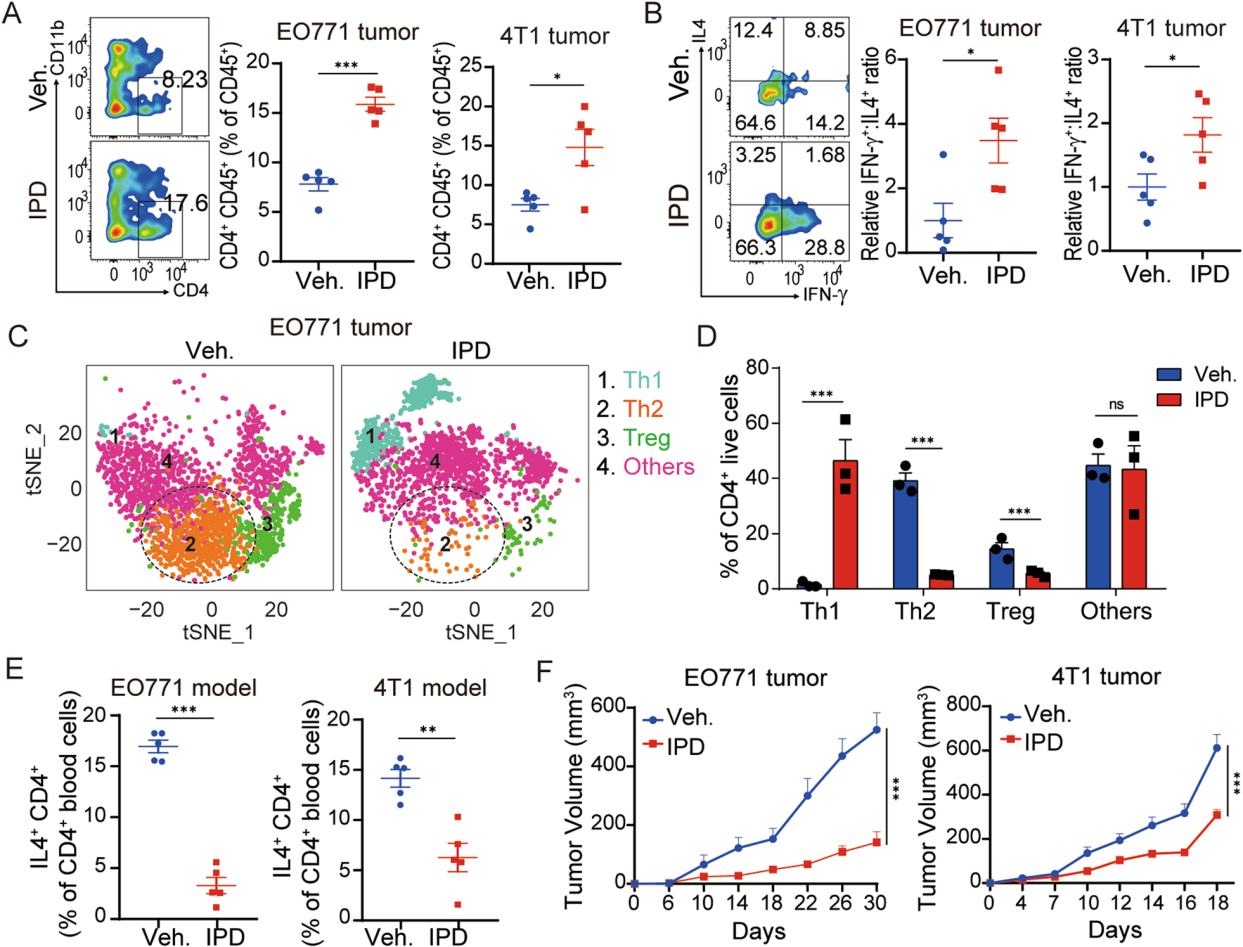
Blocking Th2-mediated immunity promotes Th2 to Th1 switch and inhibits tumor growth in breast cancer. **A** Representative contour plots and quantification of CD45^+^CD4^+^ ratios in EO771 and 4T1 tumor model (n = 5, t test). **B** Relative IFN-γ:IL-4 ratio of CD4^+^ T cells isolated from EO771 and 4T1 tumors treated with vehicle or IPD treatment (n = 5, t test). **C** T-distributed stochastic neighbor embedding (t-SNE) plot of tumor-infiltrating CD4^+^ T cells overlaid with color-coded clusters in EO771 tumors. **D** Frequency of clusters of indicated CD4^+^ T cells subsets (n = 3, t test). **E** Quantification of Th2 cell in peripheral blood cells in EO771 and 4T1 tumor models (n = 5, t test). **F** EO771 and 4T1 tumor growth in vehicle-treated versus IPD-treated mice (n ≥ 4, two-way ANOVA). Mean ± SEM; * *p* < 0.05; ** *p* < 0.01; *** *p* < 0.001; ns, not significant

We next assessed whether inhibiting Th2-mediated immunity affected breast cancer progression using the Th2 cytokine inhibitor IPD. Firstly, the effect of IPD on Th1 and Th2 differentiation in the tumors of three syngeneic mouse breast cancer models (EO771, 4T1, and EMT6 cell lines) was detected by flow cytometry. We noticed that IPD markedly increased the infiltration of total CD4^+^ T cells in tumor tissues (Fig. 2A and Additional file 2: Fig. S2A). To be noted, IPD significantly enhanced Th1 (IFN-γ^+^CD4^+^) cell infiltration and inhibited Th2 (IL-4^+^CD4^+^) cell infiltration, indicating IPD promoted severe shift from Th2 to Th1 (Fig. 2B and Additional file 2: Fig. S2B-D). The similar results were also confirmed by CyTOF (Fig. 2C, D). Furthermore, the alteration of Th2 cells in peripheral blood was also investigated. Consistently, flow cytometric analysis showed that IPD dramatically reduced circulating Th2 subpopulation (Fig. 2E and Additional file 2: Fig. S2E). Collectively, these data supported that IPD indeed inhibited Th2 cell infiltration in breast cancer. Next, we evaluated the effect of IPD on breast cancer growth in vivo. We found IPD significantly suppressed tumor growth in the three immunocompetent mouse models (Fig. 2F and Additional file 2: Fig. S2F). However, cell proliferation assay suggested that the tumor cell proliferation was not affected by IPD in vitro (Additional file 3: Fig. S3A, B), while transwell migration assay also showed no significant change about metastatic potential of tumor cells after IPD treatment (Additional file 3: Fig. S3C). These observations indicated that IPD did not directly affect tumor cells and might exert the antitumor efficacy by regulating tumor immunity.

### Inhibiting Th2-mediated immunity enhances the antitumor activity of cytotoxic CD8^+^ T cells in breast cancer

**Fig. 3:**
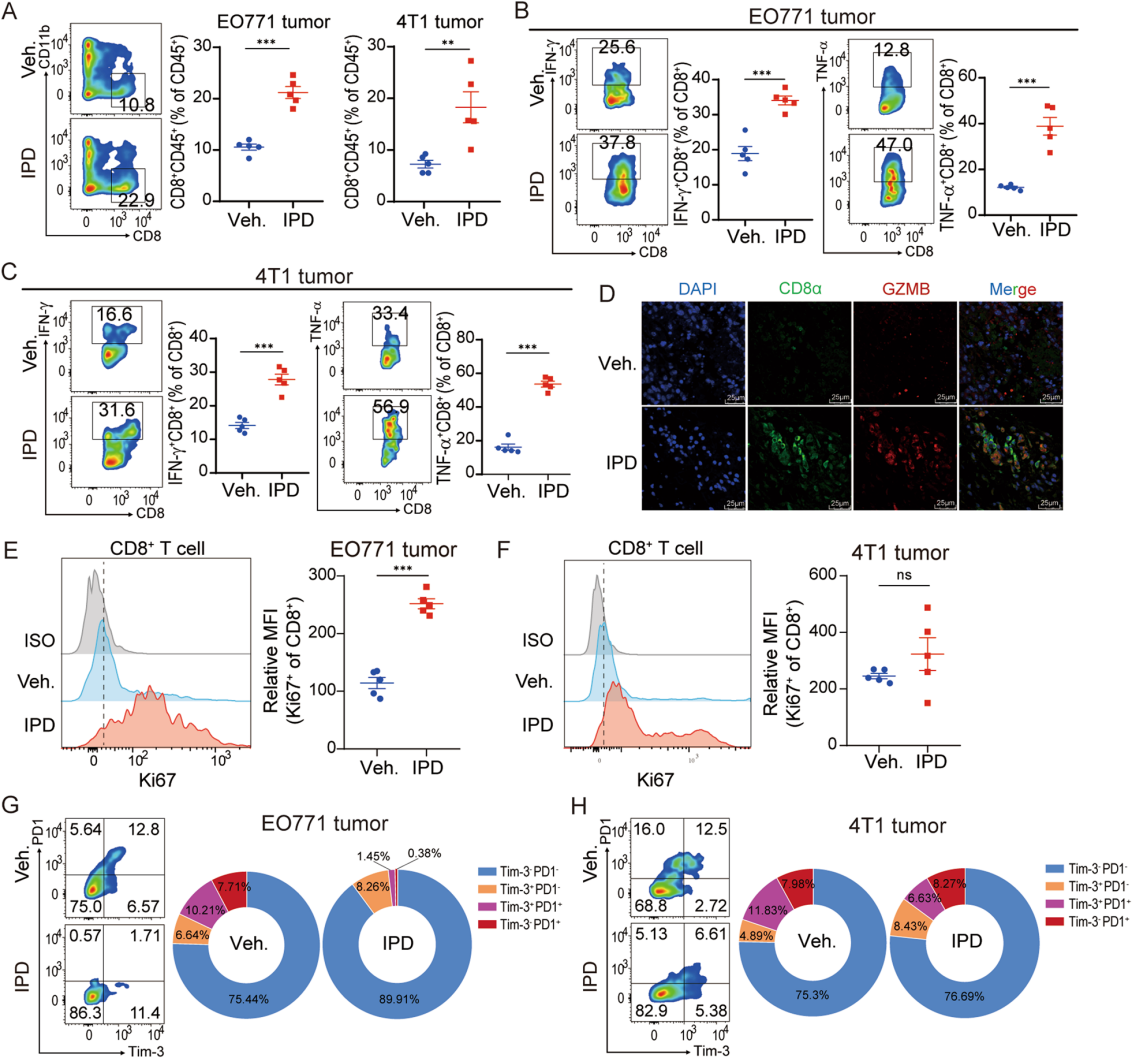
IPD enhances the antitumor activity of cytotoxic CD8^+^ T cells in breast cancer. **A** Representative contour plots and quantification of CD8^+^ T cells ratios in EO771 and 4T1 tumors (n = 5, t test). **B**, **C** Flow cytometry analysis of IFN-γ^+^ and TNF-α^+^ CD8^+^ T cells from EO771 tumor (B) and 4T1 tumor (C) (n = 5, t test). **D** Representative immunofluorescence images of CD8^+^ T cells (green) and granzyme B (red) in EO771 tumors. Scale bar, 25 μm. **E**, **F** Representative histogram of Ki-67 expression and percentages of Ki-67^+^ CD8^+^ cells in EO771 (E) and 4T1 (F) tumors (n = 5, t test). (G and H) Representative contour plots of PD-1^+^ Tim-3^+^ CD8^+^ T cells of EO771 (G) and 4T1 (H) tumors. Mean ± SEM; ** *p* < 0.01; *** *p* < 0.001; ns, not significant

Given that CD8^+^ T cells play a central role in mediating antitumor immunity, we next analyzed the effect of IPD on the infiltration and cytotoxic activities of CD8^+^ T cells [39, 40]. It is demonstrated that CD8^+^ T cell infiltration in breast cancer tissues and circulating CD8^+^ T cells significantly increased after IPD treatment (Fig. 3A and Additional file 4: Fig. S4A, B). The release of effector cytokines including IFN-γ and TNF-α from CD8^+^ T cells was also enhanced by IPD in tumors (Fig. 3B, C and Additional file 4: Fig. S4C, D). The above findings were further validated by multiplex IF staining which showed more CD8^+^ T cell infiltration and granzyme B release in IPD-treated group than in vehicle-treated group (Fig. 3D). Furthermore, the infiltrating CD8^+^ T cells displayed higher ki67 expression in IPD-treated group compared with vehicle-treated group, indicating that CD8^+^ T cells had been activated to exert antitumor activities in response to IPD treatment (Fig. 3E, F and Additional file 4: fig. S4E). T cell exhaustion is a state of T cell dysfunction that arises during cancer [41]. We found that the percentage of exhausted CD8^+^ T cells (PD-1^+^TIM3^+^) was lower in IPD-treated group compared to vehicle-treated group as determined by flow cytometry analysis, suggesting IPD effectively reversed the exhausted state of CD8^+^ T cells (Fig. 3G, H and Additional file 4: fig. S4F). To summarize, these findings suggested that IPD treatment markedly improved the antitumor efficacy of CD8^+^ T cells in breast cancer.

### Inhibiting Th2-mediated immunity reshapes the landscape in the TME

**Fig. 4:**
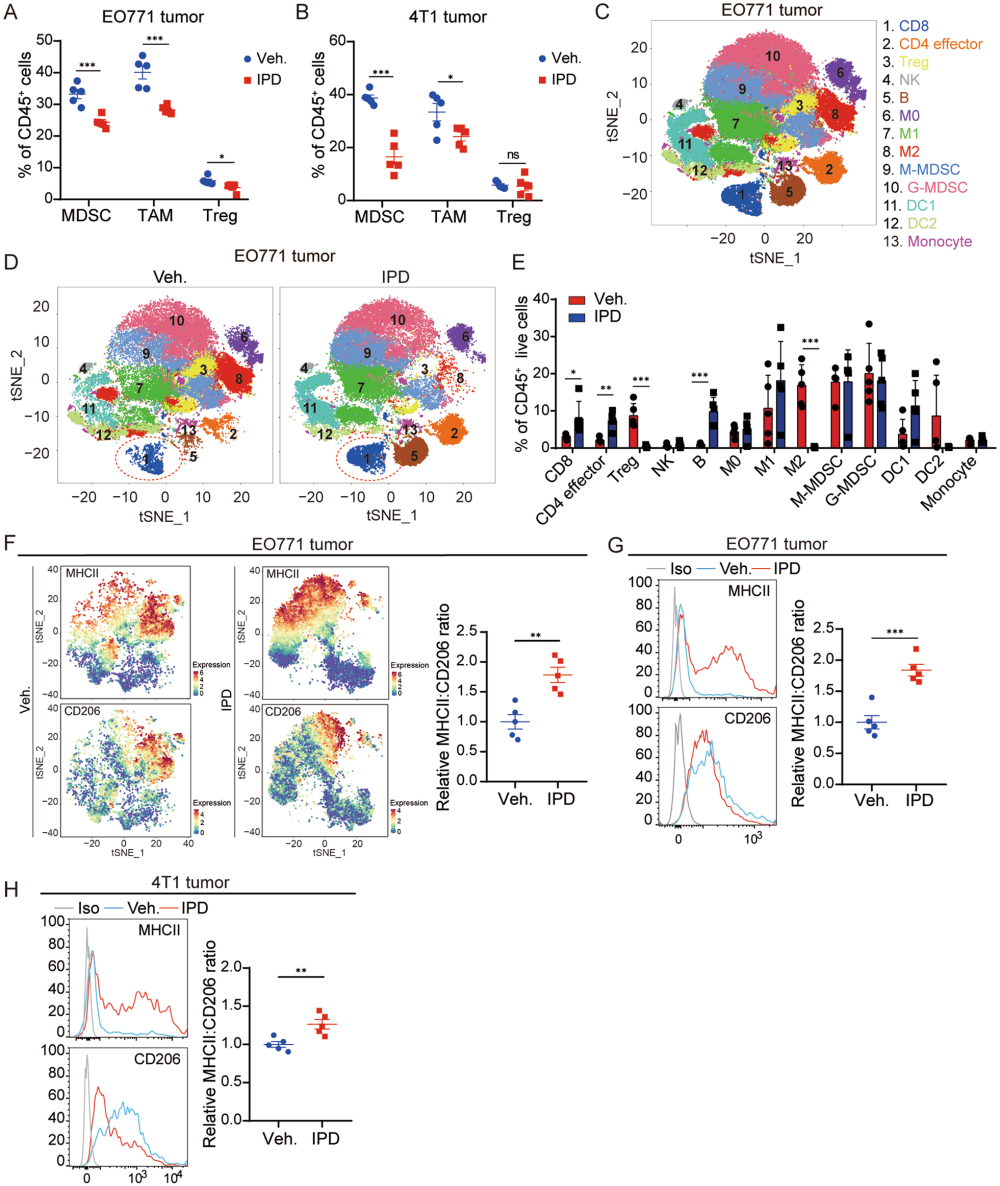
Inhibiting Th2-mediated immunity reshapes the immune landscape in the TME. **A**, **B** Representative quantification of myeloid-derived suppressor cell (MDSC), tumor-associated macrophage (TAM), and regulatory T cell (Treg) of CD45^+^ live cells in EO771 (A) and 4T1 (B) tumors (n = 5, two-way ANOVA). **C** T-SNE plot of TILs overlaid with color-coded clusters in EO771 tumors. **D** T-SNE plot of tumor-infiltrating leukocytes overlaid with color-coded clusters from vehicle-treated or IPD-treated mice. **E** Frequency of clusters of indicated immune cell subsets from vehicle-treated and IPD-treated mice (n = 5, t-test). **F** Macrophage-associated density t-SNE plots and quantification of an equal number of CD45^+^ tumor-infiltrating leukocytes in vehicle-treated versus IPD-treated mice. **G**, **H** Relative MHC II:CD206 ratio of TAMs in EO771 and 4T1 tumors from vehicle-treated versus IPD-treated mice (n = 5, t test). Mean ± SEM; * *p* < 0.05; ** *p* < 0.01; *** *p* < 0.001; ns, not significant

To deeply elucidate the effect of IPD on tumor immunity, we next investigated the alteration of tumor immune landscape after IPD treatment by flow cytometry. We found that immune-suppressive cell population MDSCs was significantly decreased in IPD-treated groups of three mouse breast cancer models, compared with vehicle-treated groups. Additionally, the infiltration of TAMs and Tregs was also markedly inhibited by IPD in EO771 and EMT6 models (Fig. 4A, B and Additional file 5: fig. S5A). To evaluate the general impact of IPD on the tumor immune microenvironment, we profiled CD45^+^ immune cells isolated from EO771 tumors grown in vehicle- and IPD-treated mice using CyTOF, which revealed 13 distinct cell clusters (Fig. 4C–E, and Additional file 5: fig. S5B–D). EO771 tumors from IPD-treated mice had significantly increased CD8^+^ T cells (cluster 1), effector CD4^+^ T cells (cluster 2) and B cells (cluster 5) and fewer M2-like macrophages (cluster 8), suggesting enhanced antitumor immunity by IPD. Th2 cells have been shown to induce the differentiation of M2 macrophages through the release of IL-4 [42]. The expression ratio of major histocompatibility complex class II (MHCII, an M1 marker) versus CD206 (an M2 marker) was used to evaluate M1/M2 polarization status of macrophages, which generally represents antitumor versus protumor activities of TAMs [26] We validated that the MHCII:CD206 ratio of TAMs was increased in IPD-treated groups by CyTOF and flow cytometry, indicating IPD could induce M2 to M1 polarization of TAMs (Fig. 4F–H and Additional file 5: fig. S5E).

### Targeting Th2-mediated immunity synergizes with ICB

**Fig. 5:**
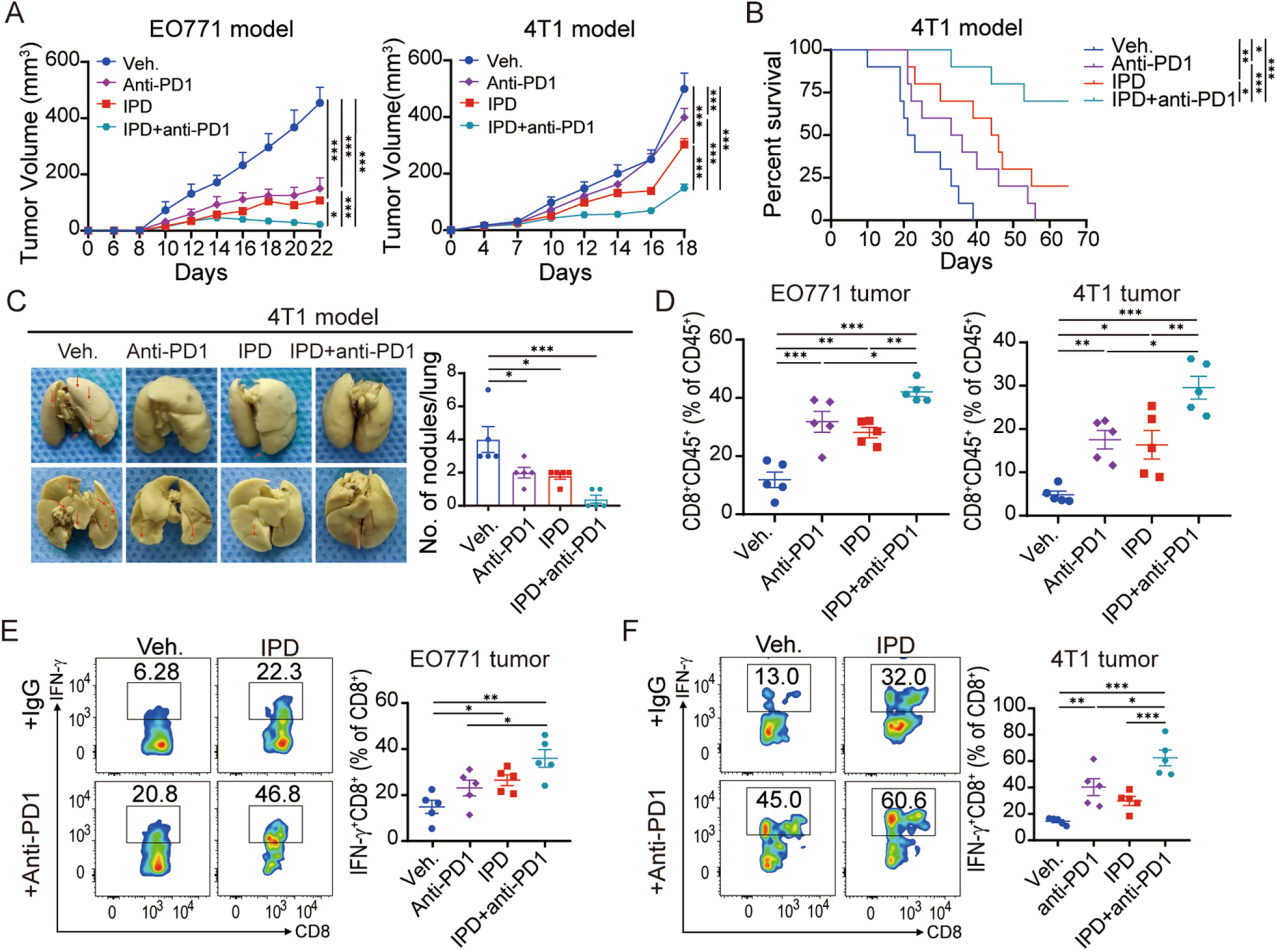
IPD synergizes with anti-PD1 treatment in breast cancer. **A** EO771 (left) and 4T1 (right) tumor growth treated with vehicle, IPD, anti-PD1, or IPD + anti-PD1 (n = 5, two-way ANOVA). **B** Kaplan-Meir survival analysis of 4T1 tumor-bearing mice with indicated treatments (n = 10, log-rank test). **C** Lung metastatic nodules from 4T1 tumor-bearing mice treated with indicated treatments (n = 5, one-way ANOVA). **D** Quantification of CD8^+^ T cells ratios in EO771 and 4T1 tumors treated with indicated treatments (n = 5, one-way ANOVA). **E**, **F** Representative flow cytometry images and percentages of IFN-γ^+^ CD8^+^ T cells from EO771 (E) and 4T1 (F) tumors (n = 5, one-way ANOVA). Mean ± SEM; * *p* < 0.05; ** *p* < 0.01; *** *p* < 0.001

**Fig. 6:**
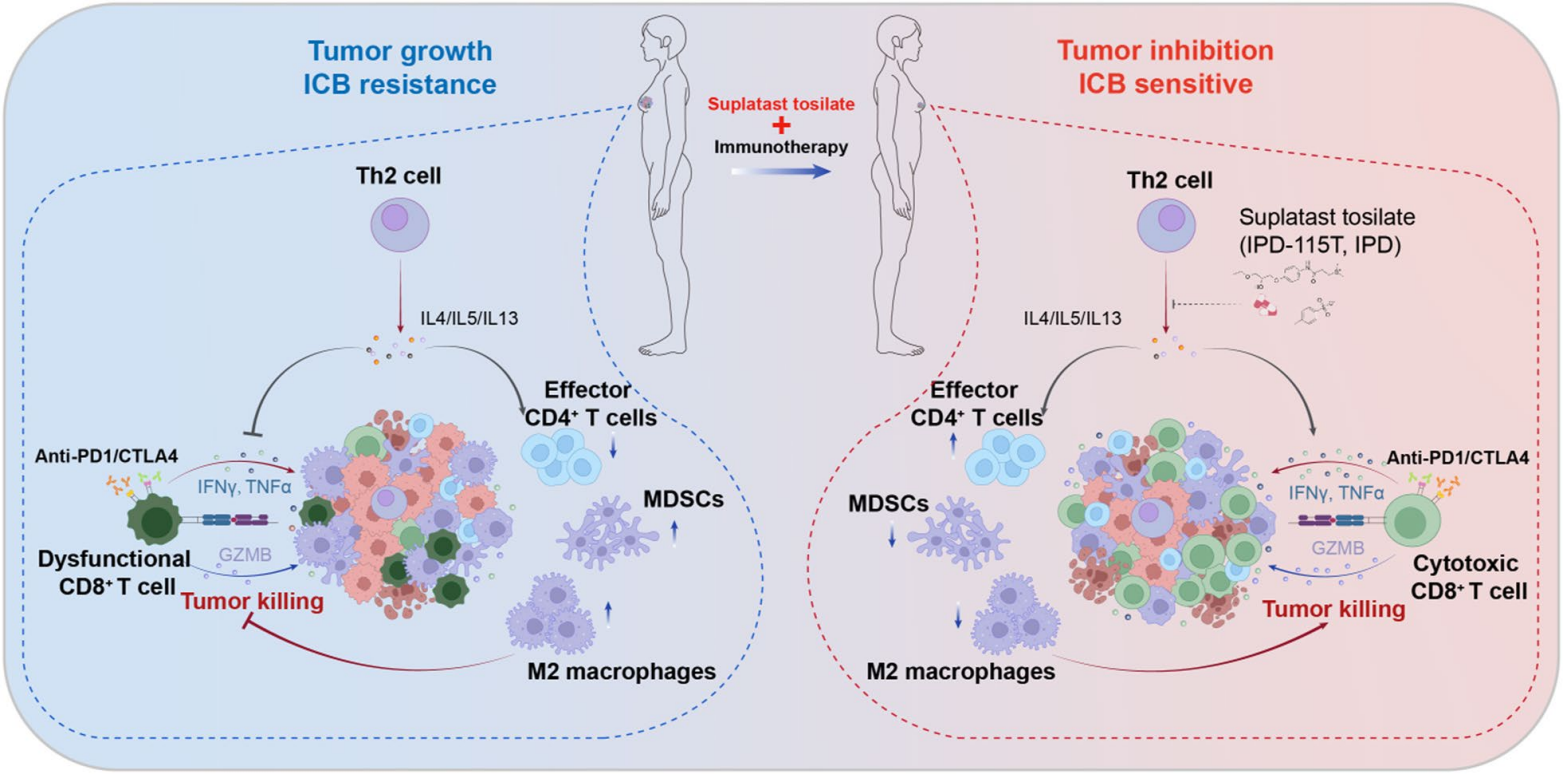
Schematic illustration of the mechanism by which IPD enhances immunotherapy response. Th2-mediated immunity suppresses ICB response by inhibiting the infiltration and cytotoxic activity of CD8^+^ T cell and reshaping the global immune landscape including decreasing the infiltration of effector CD4^+^ T cell and recruiting immunosuppressive cells such as MDSCs and M2-macrophage in tumor microenvironment. Targeting Th2 immunity by IPD can reverse the immunosuppressive microenvironment, thereby improve ICB response and inhibit tumor growth of breast cancer

To test if inhibition of Th2-mediated immunity would enhance antitumor activity of ICB, the combinatorial treatments of IPD plus ICB (anti-PD-1 or anti-CTLA-4) were used in EO771, 4T1 and EMT6 models. Single treatment with IPD or ICB therapy delayed tumor growth, and combinatorial treatment more effectively inhibited tumor growth in all three mouse models (Fig. 5A and Additional file 6: fig. S6A). Moreover, compared with anti-PD1 treatment, the combination therapy dramatically prolonged mice survival in 4T1 tumor model (Fig. 5B). 4T1 lung metastases were also significantly less in combinatorial treatment group, compared with other groups, indicating that inhibiting Th2-mediated immunity could enhance anti-metastasis immune response in vivo (Fig. 5C). Consistently, further flow cytometry analysis showed that the combination of IPD and ICB significantly enhanced the infiltration and antitumor activity of CD8^+^ T cells, compared with ICB or IPD alone (Fig. 5D-F and Additional file 6: fig. S6B, C). Total CD4^+^ T cell was further increased and immunosuppressive cells including MDSC and TAM were further decreased after the combination treatment (Additional file 6: fig. S6D-F), suggesting IPD treatment combined with ICB drastically reversed the immunosuppressive microenvironment (Fig. 6).

Considering adverse effects may cause intolerance in therapy procedures and negatively affect its clinical efficacy, we next evaluated the adverse effects of IPD in the mouse models [43, 44]. The H&E staining showed that solid organ injury of breast cancer was not enhanced after IPD treatment. Compared with ICB therapy, IPD would not increase the immune-related adverse events associated with anti-CTLA-4 in combination therapy (Additional file 7: fig. S7A). Moreover, compared with vehicle-treated group, IPD treatment did not exacerbate the liver damage and impair renal and cardiac functions, and had no haematotoxin effect and bone marrow suppression based on blood test (Additional file 7: Fig. S7B, C). Together, our results implied that related adverse event was no presented after administration of IPD in vivo.

## Discussion

The efficacy of ICB therapy has been widely evaluated in breast cancer. Unfortunately, most clinical research shows that cancer patients get limited benefit from ICB [45]. Thus, increasing combination therapies are evaluated to surmount the resistance to immunotherapy and improve clinical efficacy [46, 47]. Accumulating evidences indicate that the immunosuppressive TME inhibits ICB response and are commonly associated with poor prognosis of breast cancer patients [48]. Therefore, to explore potential therapies targeting TME might help reverse immunotherapy resistance. Th2 cells are an important immune component in TME [49, 50]. Several evidence showed that Th2 cell infiltration in TME could facilitate tumor growth [51]. The complex cytokine cross-talk of Th2 cell in TME was correlated with worse prognosis in cancer [52]. Here, our study found a strong negative correlation between Th2 cell infiltration and the infiltration and function of CD8^+^ T cells in breast cancer, suggesting that Th2 cells might be an obstacle for antitumor immunity. Consistently, targeting IL-4 and IL-5 antibodies was reported to suppress tumor progression in multiple cancers, showing targeting Th2 cytokines might be a potential therapy for tumor treatment [53–55]. However, Th2-mediated immunity is a complicated process with multiple cytokines involved. The efficacy of targeting single cytokine remains limited. Therefore, more effective agents targeting Th2-mediated immunity were also needed for investigation.

IPD, an immunoregulatory compound, has the potential to treat anaphylactic diseases [56]. Increasing studies show that IPD inhibits the secretion of Th2 cell-derived IL-4 and IL-5 and is thus defined as a Th2 cytokine inhibitor [56, 57]. Here, we demonstrated that IPD indeed increased the ratio of Th1/Th2 cells in breast cancer and suppressed the secretion of IL-4 in both peripheral blood and tumors. T cell infiltration and activation in tumor immune microenvironment are important for antitumor effects in breast cancer [11]. It is demonstrated that Th2 cells promote tumor growth by affecting T cell infiltration [58]. Our data validated that targeting Th2-mediated immunity by IPD could significantly activate the host immune response and inhibit tumor growth in breast cancer. IPD could remarkably increase the infiltration and proliferation of cytotoxic CD8^+^ T cells, and enhance the cytotoxic cytokine secretion of CD8^+^ T cells, indicating that antitumor T cell immunity was activated when Th2 cytokines were inhibited. Additionally, accumulating evidence suggests that targeting macrophages has emerged as a potential approach against ICB resistance [59, 60]. It has been acknowledged that Th2 cells induce macrophages polarized to pro-tumor M2 phenotype through the secretion of IL-4 and IL-13 [42]. Our study found that targeting Th2-mediated immunity by IPD could polarize TAMs to antitumor M1 macrophage, suggesting that blocking Th2-mediated immunity could alleviate TAMs-mediated immunosuppression. Moreover, Th2-type immune responses are usually accompanied by an increase in the infiltration of MDSCs and Tregs which contributes to pro-tumor immunity [61]. We also demonstrated that IPD decreased the infiltration of MDSCs and Tregs in breast cancer. In conclusion, targeting Th2-mediated immunity by IPD elicited an antitumor immune response against breast cancer via modulating the global immune landscape.

Furthermore, our study here showed that IPD targeting Th2-mediated immunity could enhance ICB response via promoting antitumor activity of cytotoxic CD8^+^ T cells in breast cancer. In addition, the global immune landscape was also reshaped after combinational therapy. The total CD4^+^ T cell infiltration was increased, and MDSCs and TAMs were further decreased in combinational therapy compared with single drug treatment and control group. Notably, apart from the suppression of IPD on tumor growth in vivo, we found that IPD treatment alone inhibited lung metastasis of tumor cells compared with vehicle-treated group in 4T1 spontaneous metastasis mouse model, and IPD in combination with ICB further enhanced this effect. Since the transwell migration assay showed no significant change about metastatic potential of tumor cells after IPD treatment*,* we speculated that IPD might exert the inhibitory effect on lung metastasis by regulating tumor immune environment of primary or metastatic tumors. Our previous study demonstrated that targeting histamine/HRH1 axis only partially reversed allergy-induced immunotherapy resistance [26]. This study supported that Th2-mediated immunity might also contribute to allergy-induced immunotherapy resistance and targeting Th2-mediated immunity would help reverse this resistance, which warrants further investigation. In addition, immune-related adverse event happens frequently in ICB therapy and affects healthy tissues in the body [43, 44], which is an essential factor to limit its clinical application. Here, we found that IPD had been well tolerated and would not exacerbate systemic damage of ICB in mouse models. Together, our findings implied that targeting Th2-mediated immunity might be a promising therapeutic approach in combination with ICB in clinical therapies.

## Conclusions

Our study confirms that pharmaceutical inhibition of Th2-mediated immunity by IPD could elicit an antitumor immune response against breast cancer by enhancing the antitumor activity of cytotoxic CD8^+^ T and modulating the global immune landscape of TME. Furthermore, targeting Th2-mediated immunity by IPD enhances therapeutic responses to ICB, which warrants prospectively exploring therapeutic drugs as adjuvant agents for combinatorial immunotherapy.

## Supplementary Information

**Additional file 1. Figure S1** Correlations between Th2 cell proportion and T cell infiltration and dysfunction in TCGA database.

**Additional file 2. Figure S2** IPD increases the infiltration of CD4^+^ T cells and promotes Th2 to Th1 switch.

**Additional file 3. Figure S3** IPD does not regulate the proliferation and migration of tumor cells in vitro.

**Additional file 4. Figure S4** IPD enhances the antitumor effect of CD8^+^ T cells.

**Additional file 5. Figure S5** IPD reshapes the immune landscape in the TME.

**Additional file 6. Figure S6** IPD synergizes with anti-CTLA-4 treatment in breast cancer.

**Additional file 7. Figure S7** IPD has no significant adverse reaction.

**Additional file 8. Figure S8** Gating strategy for flow cytometry assays.

**Additional file 9. ** Additional materials and methods.

## Data Availability

Publicly available datasets were analyzed in study. These can be found in the Cancer Genome Atlas (https://portal.gdc.cancer. gov/) and Cibioportal (https://www.cbioportal.org).

## Abbreviations

ICB: Immune checkpoint blockade
Th2: T-helper 2
TME: Tumor microenvironment
IPD: Suplatast tosilate
CTL: Cytotoxic T lymphocyte
TNBC: Triple-negative breast cancer
HR: Hormone receptor
ER: Estrogen receptor
PR: Progesterone receptor
TILs: Tumor-infiltrating lymphocytes
PD-1: Programmed death 1
PD-L1: Programmed death ligand 1
CTLA-4: Cytotoxic T lymphocyte-associated antigen 4
MDSC: Myeloid-derived suppressor cell
TAM: Tumor-associated macrophage
IL-4: Interleukin-4
TCGA: The Cancer Genome Atlas
METABRIC: Molecular Taxonomy of Breast Cancer International Consortium
GEO: Gene Expression Omnibus
Fges: Functional gene expression signatures
TIMER: Tumor Immune Estimation Resource
TIDE: Tumor Immune Dysfunction and Exclusion
IF: Immunofluorescence
CyTOF: Mass cytometry
Treg: Regulatory T cells
MHCII: Histocompatibility complex class II
NK cells: Natural killer cells
