# Supplementary material for: Pharmaceutical targeting Th2-mediated immunity enhances immunotherapy response in breast cancer
Source: J Transl Med. 2022 Dec 23;20:615. doi: 10.1186/s12967-022-03807-8 (PMC9783715; doi:10.1186/s12967-022-03807-8)
Supplement: Supplementary file 1 — Additional file 1. Figure S1 Correlations between Th2 cell proportion and T cell infiltration and dysfunction in TCGA database. [file 12967_2022_3807_MOESM1_ESM.docx]

**Additional figure legends.**

**
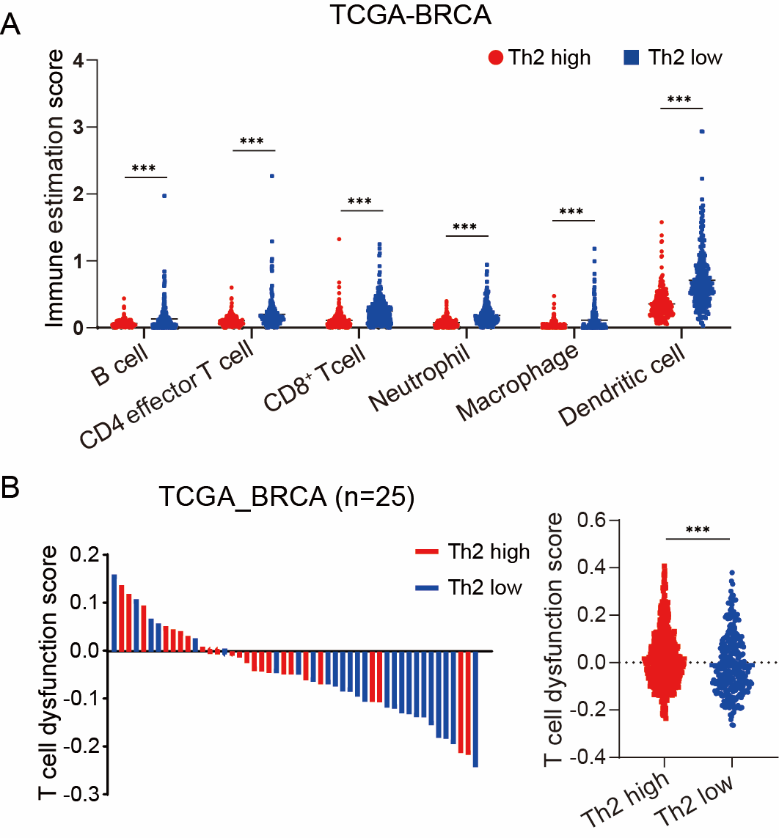
**

**Additional fig. S1** **Correlations between Th2 cell proportion and T cell infiltration and dysfunction in TCGA database.** (A) Enrichments for the indicated immune cell populations in Th2-high versus Th2-low samples in TCGA database based on TIMER (n=256, two-way ANOVA). (B) T cell dysfunction scores of Th2 cell-high and -low infiltration in breast cancer assessed by TIDE (n≥276, t test). Mean ± SEM; *** *p*<0.001.
