# Supplementary material for: Pharmaceutical targeting Th2-mediated immunity enhances immunotherapy response in breast cancer
Source: J Transl Med. 2022 Dec 23;20:615. doi: 10.1186/s12967-022-03807-8 (PMC9783715; doi:10.1186/s12967-022-03807-8)
Supplement: Supplementary file 2 — Additional file 2. Figure S2 IPD increases the infiltration of CD4+ T cells and promotes Th2 to Th1 switch. [file 12967_2022_3807_MOESM2_ESM.docx]

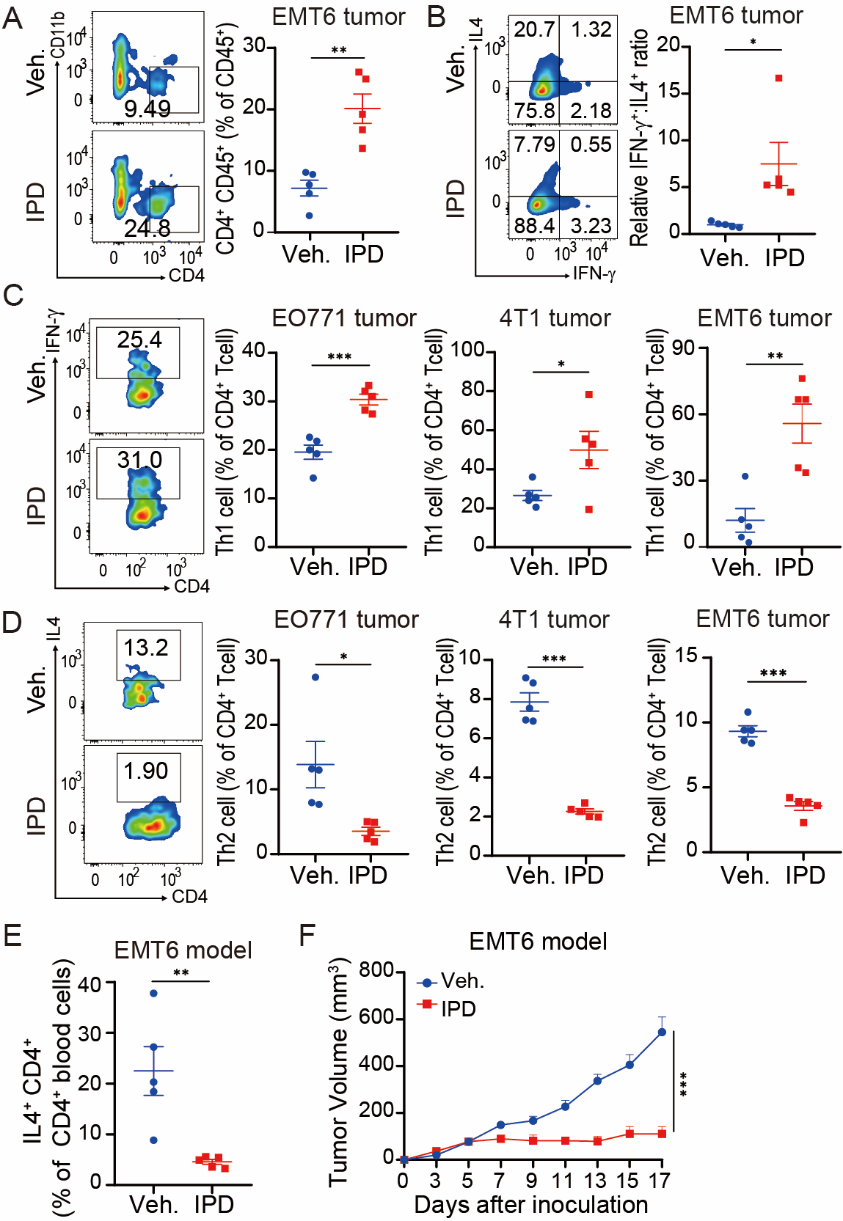


**Additional fig. S2 IPD increases the infiltration of CD4^+^ T cells and promotes Th2 to Th1 switch.** (A) Representative contour plots and quantification of CD45^+^CD4^+^ ratios in EMT6 tumors (n=5, t test). (B) Relative IFN-γ:IL-4 ratio of CD4^+^ T cell in EMT6 tumors from vehicle versus IPD treatment (n=5, t test). (C) Flow cytometry analysis of tumor-infiltrating Th1 cell (IFN-γ^+^ CD4^+^) in EO771, 4T1 and EMT6 tumors (n=5, t test). (D) Flow cytometry analysis of tumor-infiltrating Th2 cell (IL-4^+^ CD4^+^) in EO771, 4T1 and EMT6 tumors (n=5, t test). (E) Quantification of Th2 cell (IL-4^+^ CD4^+^) in peripheral blood cells from EMT6 tumor-bearing model (n=5 t test). (F) EMT6 tumor growth in vehicle-treated versus IPD-treated mice (n=5, two-way ANOVA). Mean ± SEM; * *p*<0.05; ** *p*<0.01; *** *p*<0.001.
