# Supplementary material for: Pharmaceutical targeting Th2-mediated immunity enhances immunotherapy response in breast cancer
Source: J Transl Med. 2022 Dec 23;20:615. doi: 10.1186/s12967-022-03807-8 (PMC9783715; doi:10.1186/s12967-022-03807-8)
Supplement: Supplementary file 3 — Additional file 3. Figure S3 IPD does not regulate the proliferation and migration of tumor cells in vitro. [file 12967_2022_3807_MOESM3_ESM.docx]

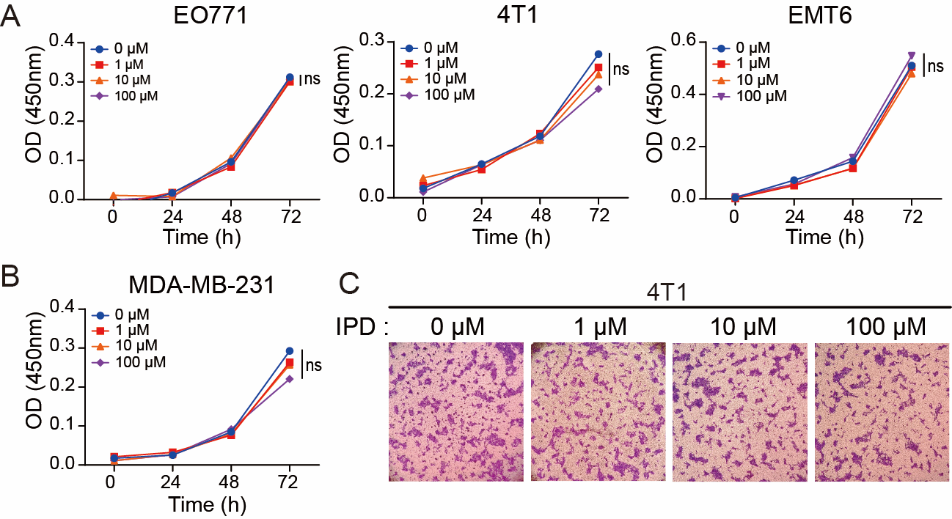


**Additional fig. S3 IPD does not regulate the proliferation and migration of tumor cells *in vitro*.** (A) CCK8 assay showing *in vitro* proliferation of EO771, 4T1 and EMT6 cells at different concentrations of IPD (n=3, two-way ANOVA). (B) CCK8 assay showing *in vitro* proliferation of MDA-MB-231 cells at different concentrations of IPD (n=3, two-way ANOVA). (C) Representative microscopic images of transwell assay (Giemsa stain) with different concentrations of IPD in 4T1 tumor cells. Mean ± SEM; ns, not significant.
