# Supplementary material for: Pharmaceutical targeting Th2-mediated immunity enhances immunotherapy response in breast cancer
Source: J Transl Med. 2022 Dec 23;20:615. doi: 10.1186/s12967-022-03807-8 (PMC9783715; doi:10.1186/s12967-022-03807-8)
Supplement: Supplementary file 4 — Additional file 4. Figure S4 IPD enhances the antitumor effect of CD8+ T cells. [file 12967_2022_3807_MOESM4_ESM.docx]

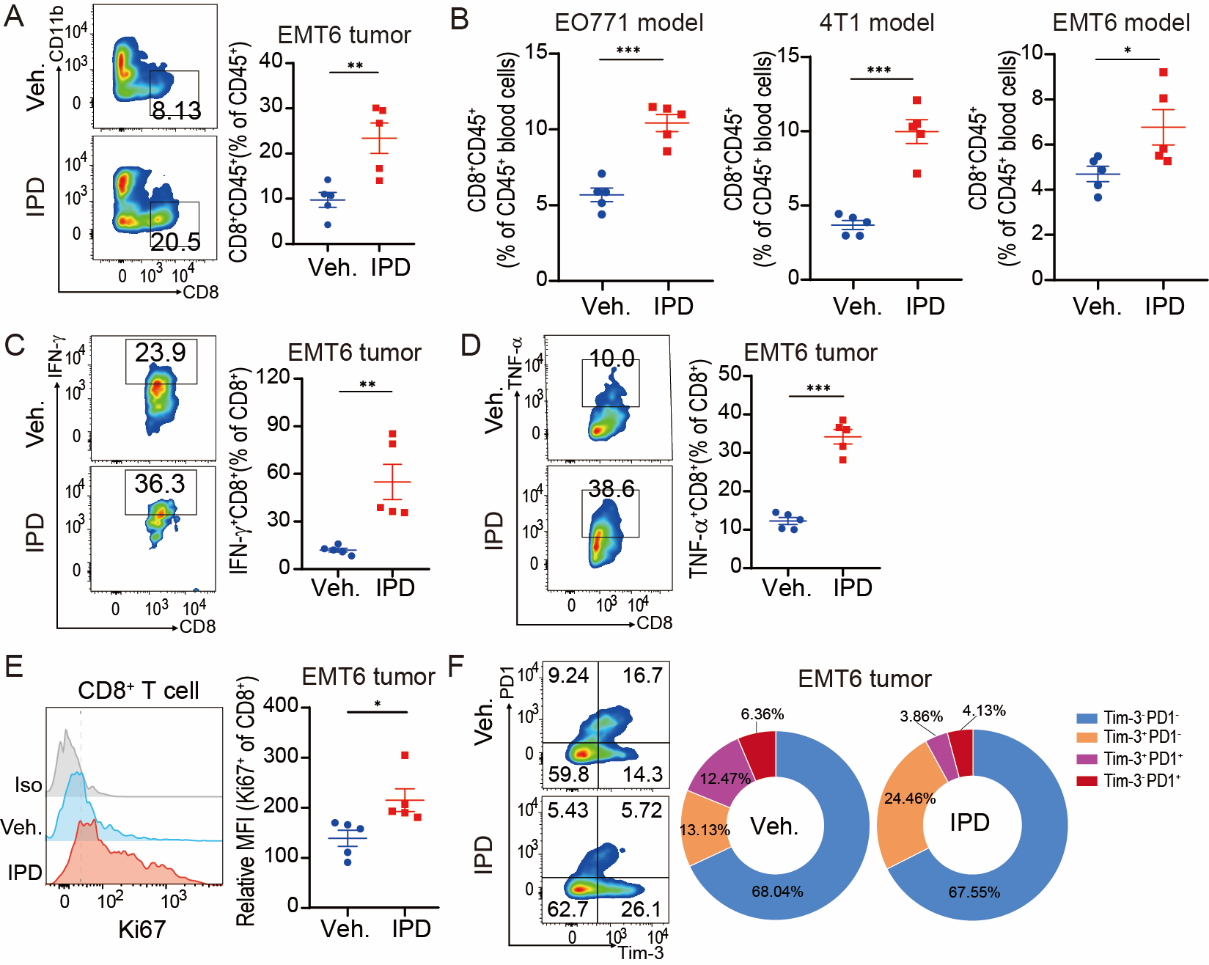


**Additional fig. 4 IPD enhances the antitumor effect of CD8^+^ T cells.** (A) Representative contour plots and quantification of CD8^+^ T cells ratios in vehicle-treated versus IPD-treated EMT6 mice (n=5, t test). (B) Quantification of CD8^+^ T cell ratios in peripheral blood cells from EO771, 4T1, and EMT6 tumor-bearing models (n=5, t test). (C and D) Flow cytometry analysis of IFN-γ^+^ CD8^+^ (C) and TNF-α^+^ CD8^+^ (D) T cells from EMT6 tumors (n=5, t test). (E) Representative histogram of Ki-67 expression and percentages of Ki-67^+^ CD8^+^ cells in EMT6 tumors (n=5, t test). (F) Representative contour plots of PD-1^+^ Tim-3^+^ CD8^+^ T cells in EMT6 tumors. Mean ± SEM; * *p*<0.05; ** *p*<0.01; *** *p*<0.001.
