# Supplementary material for: Pharmaceutical targeting Th2-mediated immunity enhances immunotherapy response in breast cancer
Source: J Transl Med. 2022 Dec 23;20:615. doi: 10.1186/s12967-022-03807-8 (PMC9783715; doi:10.1186/s12967-022-03807-8)
Supplement: Supplementary file 5 — Additional file 5. Figure S5 IPD reshapes the immune landscape in the TME. [file 12967_2022_3807_MOESM5_ESM.docx]

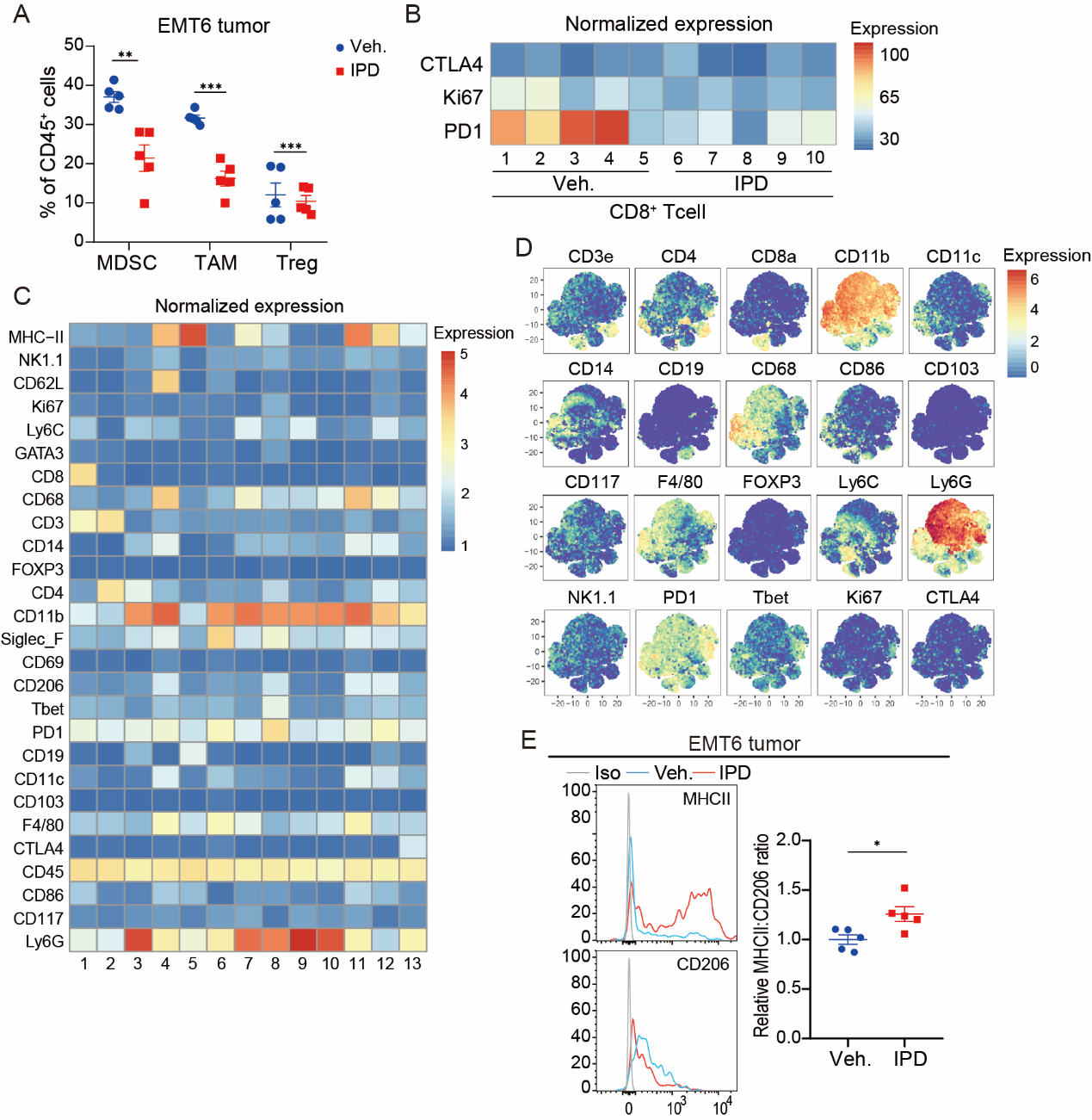


**Additional fig. S5 IPD** **reshapes the immune landscape in the TME.** (A) Representative quantification of MDSC, TAM and Treg in the EMT6 tumors (n=5, two-way ANOVA). (B) The normalized expression value of proliferation and exhaustion marker on tumor-infiltrating CD8^+^ T cells. (C) Heatmap displaying normalized marker expression of each immune cluster. (D) Density t-SNE plots of an equal number of CD45^+^ tumor-infiltrating leukocytes in vehicle-treated and IPD-treated mice. (E) Relative MHC II:CD206 ratio of TAMs in EMT6 tumors from vehicle-treated and IPD-treated mice (n=5, t test). Mean ± SEM; * *p*<0.05; ** *p*<0.01; *** *p*<0.001.
