# Supplementary material for: Pharmaceutical targeting Th2-mediated immunity enhances immunotherapy response in breast cancer
Source: J Transl Med. 2022 Dec 23;20:615. doi: 10.1186/s12967-022-03807-8 (PMC9783715; doi:10.1186/s12967-022-03807-8)

**Additional fig. S6 IPD synergizes with anti-CTLA-4 treatment in breast cancer.** (A) EMT6 tumor growth in mice treated with vehicle, IPD, anti-CTLA-4, or IPD+anti-CTLA-4 (n=5, two-way ANOVA). (B) Quantification of CD8^+^ T cells ratios in EMT6 tumors treated with indicated treatments (n=5, one-way ANOVA). (C) Representative flow cytometry images and quantification of IFN-γ^+^ CD8^+^ T cells in EMT6 tumors treated with indicated treatments (n=5, one-way ANOVA). (D, E and F) Percentages of CD4^+^ T cell, MDSC, and TAM of CD45^+^ live cells in EO771 (D), 4T1 (E), and EMT6 (F) tumors treated with vehicle, IPD, ICB (anti-PD1 or anti-CTLA-4), or IPD+ICB (n=5, one-way ANOVA). Mean ± SEM; * *p*<0.05; ** *p*<0.01; *** *p*<0.001; ns, not significant.


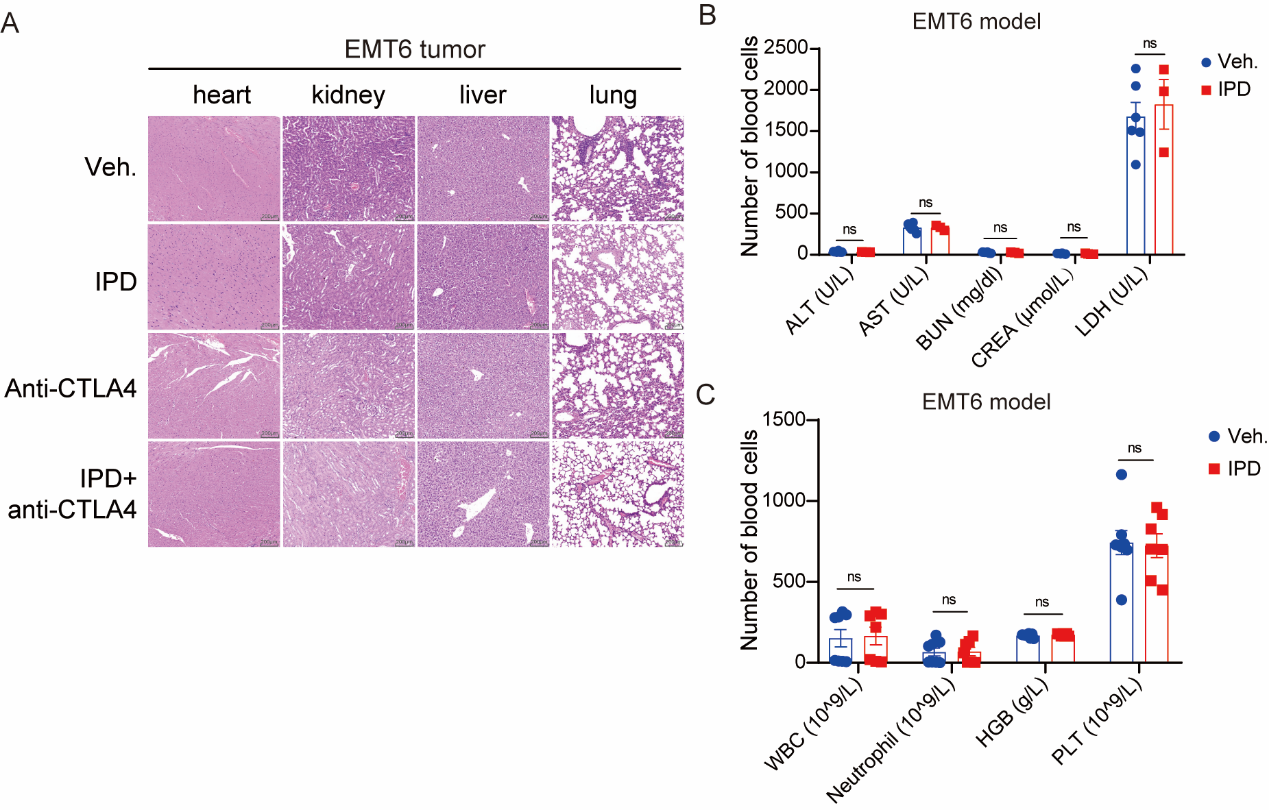

Supplement: Supplementary file 6 — Additional file 6. Figure S6 IPD synergizes with anti-CTLA-4 treatment in breast cancer. [file 12967_2022_3807_MOESM6_ESM.docx]
