# Supplementary material for: Pharmaceutical targeting Th2-mediated immunity enhances immunotherapy response in breast cancer
Source: J Transl Med. 2022 Dec 23;20:615. doi: 10.1186/s12967-022-03807-8 (PMC9783715; doi:10.1186/s12967-022-03807-8)
Supplement: Supplementary file 7 — Additional file 7. Figure S7 IPD has no significant adverse reaction. [file 12967_2022_3807_MOESM7_ESM.docx]

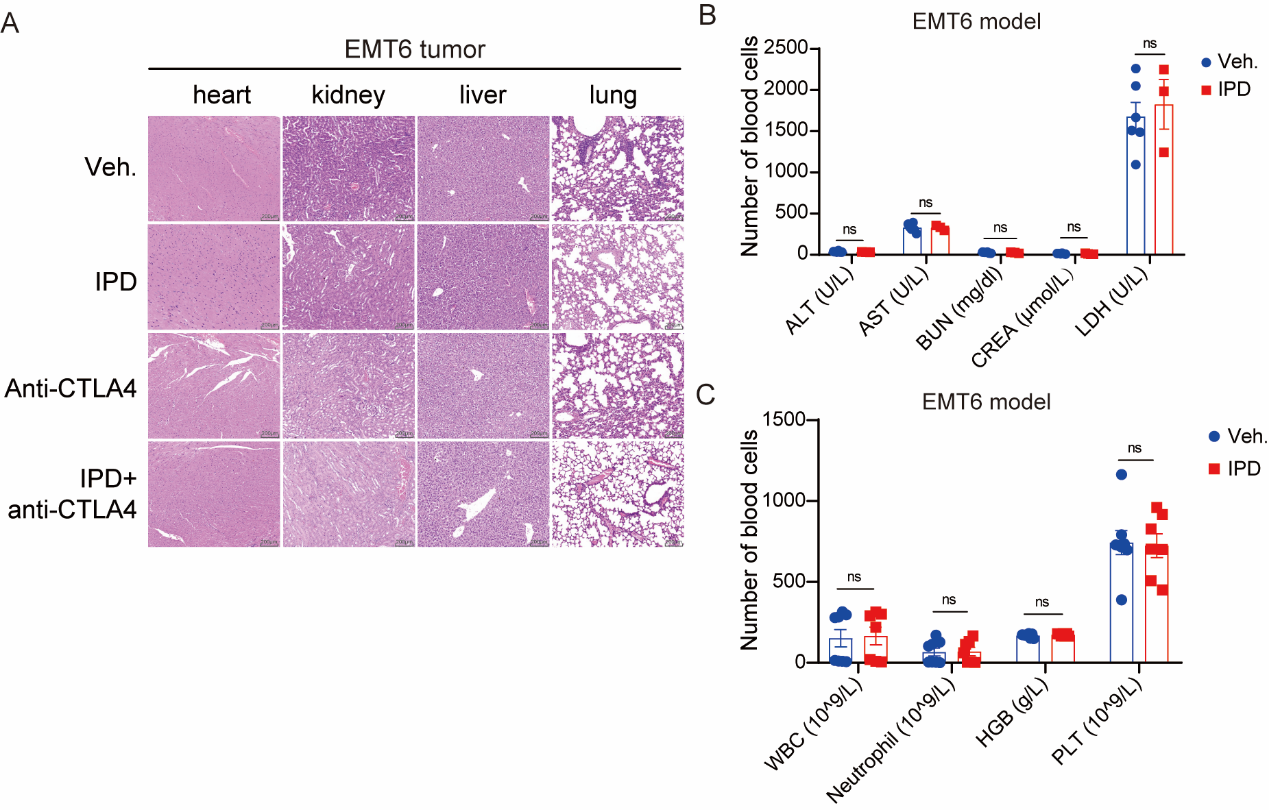


**Additional fig. S7 IPD has no significant adverse reaction.** (A) The micro pathological images (H&E staining, Magnification 20x) of tissues including heart, liver, lung, and kidney after IPD treatment in EMT6 tumor-bearing model. (B) Differences in liver and kidney function analysis in EMT6 tumor-bearing model after IPD treatment (n=6, two-way ANOVA). (C) Differences in blood routine examination analysis in EMT6 tumor-bearing model after IPD treatment (n=6, two-way ANOVA). Mean ± SEM; * *p*<0.05; ** *p*<0.01; *** *p*<0.001.
