# Supplementary material for: Pharmaceutical targeting Th2-mediated immunity enhances immunotherapy response in breast cancer
Source: J Transl Med. 2022 Dec 23;20:615. doi: 10.1186/s12967-022-03807-8 (PMC9783715; doi:10.1186/s12967-022-03807-8)
Supplement: Supplementary file 8 — Additional file 8. Figure S8 Gating strategy for flow cytometry assays. [file 12967_2022_3807_MOESM8_ESM.docx]

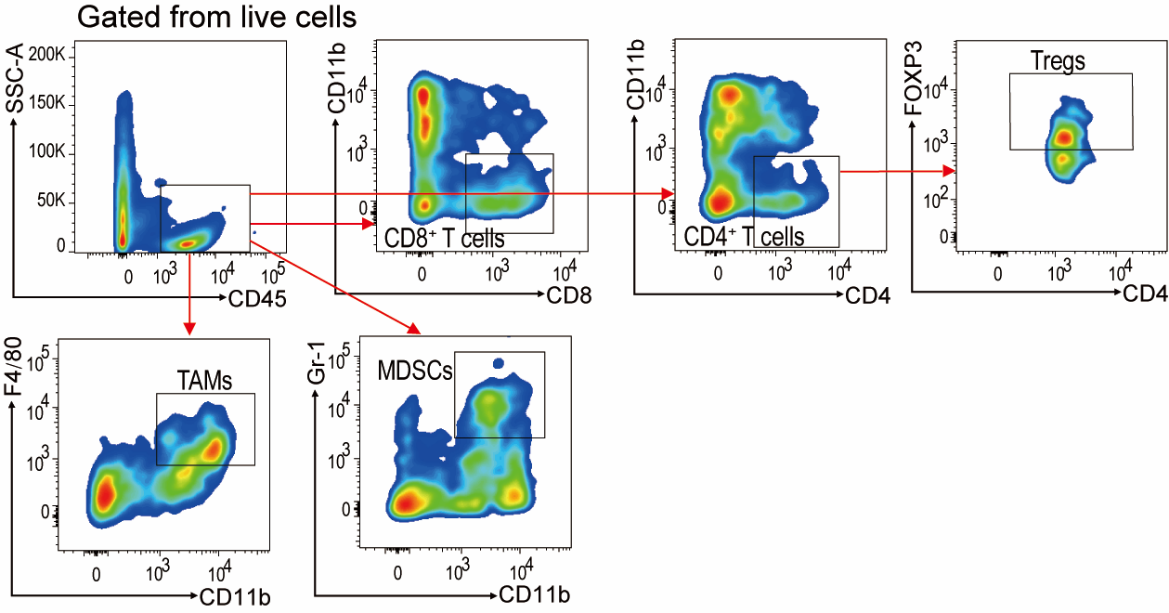


**Additional fig. S8 Gating strategy for flow cytometry assays.** Representative gating strategy used to identify CD8^+^ T cell (CD45^+^CD11b^-^CD8^+^), CD4^+^ T cells (CD45^+^CD11b^-^CD4^+^), MDSCs (CD45^+^CD11b^+^Gr-1^+^), TAMs (CD45^+^CD11b^+^F4/80^+^) and Tregs (CD45^+^CD11b^-^CD4^+^FOXP3^+^) subsets.
