# Supplementary material for: Pharmaceutical targeting Th2-mediated immunity enhances immunotherapy response in breast cancer
Source: J Transl Med. 2022 Dec 23;20:615. doi: 10.1186/s12967-022-03807-8 (PMC9783715; doi:10.1186/s12967-022-03807-8)
Supplement: Supplementary file 9 — Additional file 9. Additional materials and methods. [file 12967_2022_3807_MOESM9_ESM.docx]

**Additional materials and methods.**

**Cell proliferation assays**

MDA-MB-231, EO771, 4T1 and EMT6 cells were seeded in 96-well plates at 37 ℃ overnight. The cell growth rate was measured at 0, 24, 48 and 72 h with different concentrations (1, 10, 100 μM), using the Cell Counting Kit-8 (CCK-8; Beyotime, Shanghai, China).

**Migration assays**

The migration was performed in a 24-well plate for 24 hours respectively. 4T1 cells (2 × 10^4^) in 200 μl of serum-free medium were seeded onto the upper Cell Culture Insert with 8 μm pores (Corning) The lower chamber contained 700 μl of complete medium which included IPD with different concentrations (1, 10, 100 μM). The cells migrated to the cell culture insert membrane which was fixed with polyformaldehyde for 30 min and stained with 0.005% crystal violet for 20 min. The numbers of migrated cells were counted under the microscope from 10 random fields.
